# Current Knowledge of the Etiology and Management of Molar Incisor Hypomineralization in Children: A Narrative Review

**DOI:** 10.7759/cureus.74770

**Published:** 2024-11-29

**Authors:** Rawan Alrehaili, Ahmed Khalil, Jubarah Mergami, Almaha Koriri, Nusaybah Yamani, Shahad Albayat, Ali Alqurainiy, Bashayer Alghamdi, Nouf Alzaaqi, Ali Assiry

**Affiliations:** 1 Dentistry, Private Practice, Medina, SAU; 2 Dentistry, Ministry of Health, Najran, SAU; 3 Dentistry, Ministry of Health, Jizan, SAU; 4 Dentistry, Private Practice, Alkhobar, SAU; 5 Dentistry, Johns Hopkins Aramco Healthcare, Dahran, SAU; 6 Dentistry, Ministry of Health, Hail, SAU; 7 Dentistry, Ministry of Health, Riyadh, SAU; 8 Preventive Dental Science, Faculty of Dentistry, Najran University, Najran, SAU

**Keywords:** children, enamel defect, enamel hypomineralization, enamel hypoplasia, molar incisor hypomineraliation

## Abstract

Molar incisor hypomineralization (MIH) is a developmental condition affecting the enamel, primarily targeting one to four permanent first molars, often with the involvement of permanent incisors. The condition is characterized by distinct white-yellow or yellow-brown opacities, with more severe cases exhibiting hypomineralized enamel that is prone to breakdown. Recent data highlights MIH as a widespread dental issue seen across the globe. Despite its prevalence, the exact etiology remains unclear due to the variety of potential contributing factors. Managing MIH is particularly challenging, requiring a holistic approach to address the broad spectrum of symptoms and the heightened sensitivity of the affected teeth. Given the unique challenges of MIH, it is essential to gather updated and thorough knowledge. This understanding is critical for exploring potential preventive measures and enhancing treatment outcomes. This review aimed to examine the underlying causes of MIH, assess various treatment strategies, and offer a thorough understanding of the condition based on the latest research.

## Introduction and background

Molar incisor hypomineralization (MIH) refers to a systemic-origin hypomineralization that primarily affects one to four permanent first molars and is often accompanied by permanent incisors [1]. The European Academy of Paediatric Dentistry first defined MIH as a qualitative defect in enamel formation [2]. This condition typically presents as distinct white-yellow or yellow-brown opacities, with more severe cases showing significantly hypomineralized enamel that may be prone to breakdown [3]. In rare instances, the enamel of the affected molars deteriorates shortly after eruption, exposing the underlying dentin [4]. This condition is known as "posteruptive enamel breakdown” [5]. Teeth affected by MIH are sensitive not only to temperature changes but also to mechanical stimuli, potentially causing pain [6-8]. This discomfort can interfere with basic daily activities such as chewing capacity [9]. The food-retentive surfaces and heightened tooth sensitivity associated with MIH reduce the ability to brush effectively, which in turn compromises oral hygiene and elevates caries susceptibility [10-12]. Additionally, aesthetic burden becomes a significant concern when the incisors are involved [13].

The exact etiology of MIH remains uncertain due to the wide range of potential contributing factors. This included exposure to specific medications, such as certain antibiotics and anticonvulsants taken during pregnancy or early childhood, which have been linked to an increased risk of enamel defects [14]. Additionally, several childhood illnesses, birth-related hypoxia, and the presence of hypomineralized second primary molars have been recognized as contributing factors [15]. While the presence of hypomineralized second primary molars has been suggested as a potential indicator for increased MIH risk, its absence does not rule out the condition [16-20]. Recent research suggested that genetic predisposition, alongside epigenetic factors, may also play a role in the development of MIH [21].

Management of MIH demands a holistic approach, as it can be challenging due to the wide range of clinical symptoms and the sensitivity of the affected teeth [22]. The key objectives of MIH treatment are to alleviate sensitivity, protect the remaining enamel, and enhance esthetics [23]. Management approaches should be personalized to each patient, factoring in the condition's severity and the individual needs.

MIH is recognized for its negative effects on the psychosocial well-being of children, leading to self-consciousness and reluctance to smile [24-27]. Consequently, there is growing interest within the dental community in patient-reported outcomes, particularly in evaluating the oral health-related quality of life of children with MIH [28-31]. Given the distinct clinical challenges posed by MIH, obtaining up-to-date and comprehensive information is crucial. This knowledge is essential not only for understanding the etiology of the condition to explore potential prevention strategies but also for improving patient outcomes through effective management strategies. This narrative review aimed to explore the etiological factors contributing to MIH and evaluate various treatment options in light of the latest evidence. Additionally, it aims to shed light on the prevalence of MIH in children to offer a comprehensive understanding of the condition and its up-to-date management strategies.

## Review

Search strategy

A comprehensive literature search was performed across several established electronic databases, including PubMed, Embase, MEDLINE (Medical Literature Analysis and Retrieval System Online), and Google Scholar, to find peer-reviewed articles relevant to this review, up to June 1, 2024. The search included original research, review articles, systematic reviews, clinical trials, and animal studies, all published in English. Initially, titles and abstracts were screened for relevance, followed by a full-text review of selected articles to ensure they aligned with the review's focus. Studies in languages other than English, unrelated topics, opinion pieces, and editorials with limited relevance were excluded from consideration. 

Prevalence

The findings of multiple studies consistently highlight the global prevalence of MIH and its variation across regions and populations. Zhao et al., through an analysis of 70 studies involving 89,520 participants, reported a global MIH prevalence of 14.2%, with considerable regional differences [32]. South America exhibited the highest prevalence at 18.0%, while Africa had the lowest at 10.9%. On a country level, Spain had a prevalence of 21.1%, and India 8.1%. Their study found that children under 10 years of age had a higher prevalence (15.1%) than older children (12.1%). The authors highlighted the importance of early intervention and preventive strategies for MIH, particularly in younger populations. Similarly, Schwendicke et al. analyzed 99 studies across 43 countries and estimated a global MIH prevalence of 13.1% [33]. Their study revealed that MIH was less prevalent in high-income regions compared to areas in South Asia and Sub-Saharan Africa, where access to dental care is limited. Furthermore, the study emphasized that 27.4% of MIH cases required therapeutic intervention due to symptoms such as pain, hypersensitivity, or enamel breakdown. The authors emphasized the need for healthcare systems to consider MIH when developing policies, especially in regions with high prevalence rates and growing populations. In line with these findings, Lopes et al. analyzed data from 116 observational studies covering 50 countries and estimated the global MIH prevalence at 13.5% [34]. The highest rates were observed in the Americas at 15.3%, while Asia had the lowest prevalence at 10.7%. Notably, the study also revealed that 36.6% of MIH cases involved affected incisors, with moderate to severe cases accounting for 36.3%. 

Regarding the prevalence of MIH across genders, studies conducted by Parikh et al. [35], Preusser et al. [36], and Garcia-Margarit et al. [37] suggested a marginally higher occurrence in male children. Conversely, research by Zawaideh et al. [38] and Chawla et al. [39] indicated that MIH was more common in female children. However, other studies have reported no significant gender differences in MIH prevalence [40-43].

**Figure 1:**
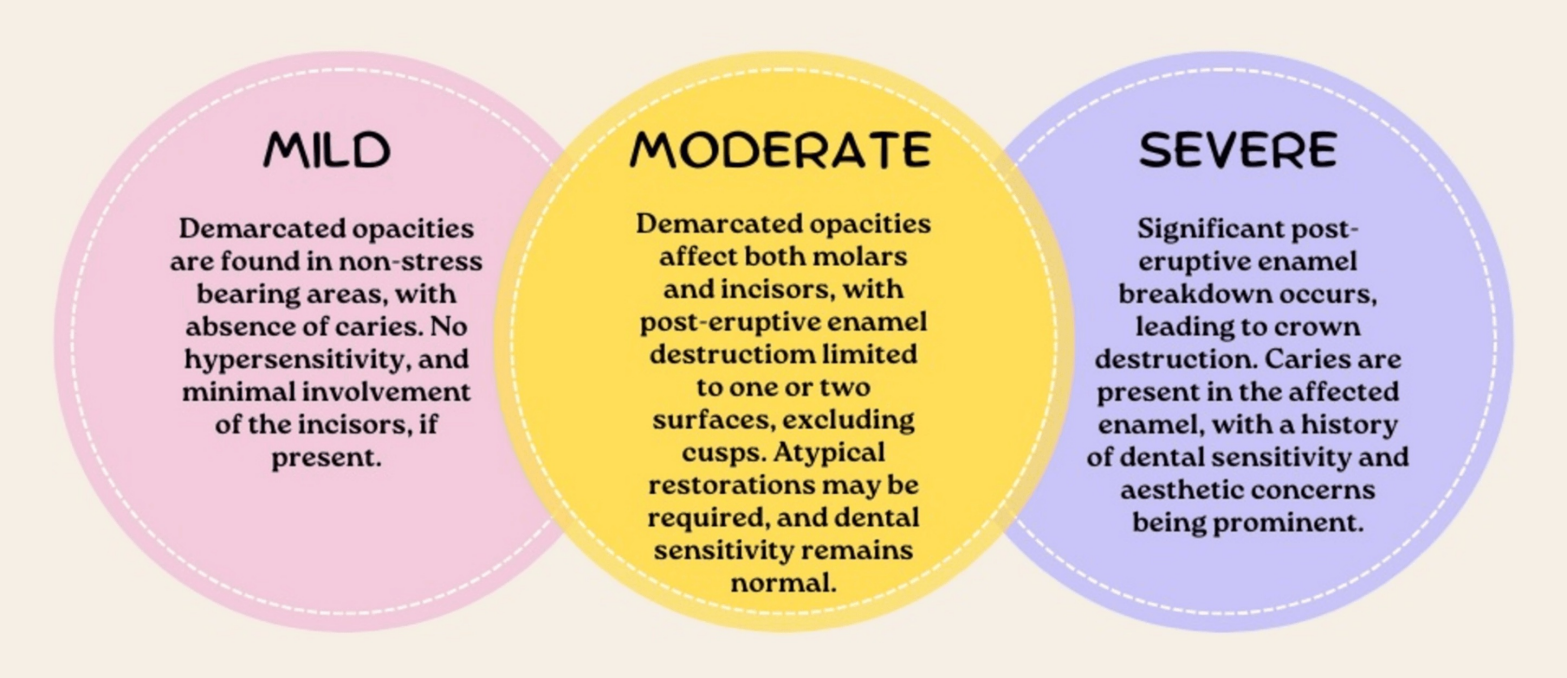
The progression of molar incisor hypomineralization severity Image Credit: Ahmed Khalil (author); created with the help of www.canva.com Reference: [44]

The progression of MIH severity according to Mathu-Muju and Wright [44] is depicted in Figure 1.

Etiology

Genetic Factors

The genetic basis of MIH has been increasingly recognized, with multiple studies identifying specific gene variants associated with the condition. Hocevar et al. investigated the potential genetic basis of MIH and identified a significant association between the rs2245803 single nucleotide polymorphism in the *MMP20* gene and the development of MIH [45]. Their findings suggested that individuals with this genetic variant, particularly in its homozygous form, had an increased susceptibility to MIH. Bussaneli et al. explored the relationship between genetic polymorphisms in immune response and amelogenesis-related genes and the development of MIH [46]. Their study identified a significant association between the rs10733708 polymorphism in the *TGFBR1* gene and severe MIH cases, suggesting that this genetic variation may increase susceptibility to MIH. Furthermore, the study highlighted gene-gene interactions, particularly between amelogenesis-related genes like AMELX and immune response genes such as IL4 and IL17A, indicating a synergistic effect that may contribute to MIH development. Teixeira et al. conducted a cross-sectional study using twins to explore the genetic and environmental factors contributing to MIH [47]. Their study found a higher concordance of MIH in monozygotic twins compared to dizygotic twins, indicating a genetic influence on the condition.

Jeremias et al. expanded on these findings by exploring the association between genetic variations in enamel formation genes and MIH [48]. Their study identified a significant link between the ENAM rs3796704 marker and the occurrence of MIH in both Brazilian and Turkish populations, suggesting a potential genetic predisposition. Additionally, the study found associations between other enamel-related genes, such as *AMELX* and *AMBN*, and MIH severity. Pang et al. conducted a case-control study investigating genetic factors contributing to MIH [49]. Their findings revealed significant associations between specific genetic polymorphisms, such as rs13115627 in the *AMBN* gene and rs1784418 in the *MMP20* gene, and the susceptibility to MIH. Additionally, the study identified an essential gene-gene interaction involving the Aquaporin 5 (*AQP5*) gene, where certain polymorphisms increased the risk of MIH. Kuhnisch et al. conducted a genome-wide association study to investigate the genetic basis of MIH [50]. The study identified a potential link between the *SCUBE1* gene, located on chromosome 22, and the presence of MIH.

These studies suggest that certain genetic variants, particularly in homozygous forms, increase susceptibility to MIH and that gene interactions may contribute to its development.

Environmental Factors

Environmental, medical, and systemic factors play significant roles in the development of MIH. Durmus et al. found that prenatal conditions such as bronchitis and hypertension did not correlate with MIH [51]. Yet, postnatal factors, particularly prolonged medication use and asthma before age three, were commonly associated with MIH. This was supported by Wogelius et al., who observed that children who had used β2-agonists and corticosteroids before age three had a higher prevalence of severe demarcated opacities, although overall MIH risk was not elevated by asthma drug use [52]. Shinde and Winnier reinforced the importance of early childhood treatments by demonstrating a strong association between aerosol therapy and MIH, showing that children exposed to aerosol therapy were twice as likely to develop MIH, with early antibiotic use further increasing the risk [53].

On the other hand, Van der Tas et al. found no significant link between vitamin D levels during fetal, neonatal, or childhood stages and MIH by age six, despite initial suggestions that lower vitamin D might contribute [54]. Costa-Silva et al. also found no significant correlation between deciduous molar hypomineralization and MIH or between common prenatal and perinatal conditions and MIH, suggesting that certain factors thought to influence enamel development might not directly contribute to MIH [55]. Kuklik et al. provided evidence that systemic conditions, such as celiac disease, significantly increased the risk of MIH, with a 4.75-fold greater likelihood of developing the condition due to malabsorption-related nutritional deficiencies [56]. Similarly, Andrade et al. found a significantly higher prevalence of MIH in HIV-infected children, particularly those treated with protease inhibitors, further highlighting the influence of systemic conditions on enamel development [57]. Hysi et al. demonstrated a strong link between respiratory illnesses and MIH, showing that children with respiratory issues and frequent antibiotic use in the first three years of life were more likely to develop MIH [58]. This was echoed by Tourino et al., who linked childhood conditions such as asthma, bronchitis, and the need for oxygenation at birth to an increased prevalence of MIH, reinforcing the idea that early respiratory conditions significantly contribute to the condition [59].

Ahmadi et al. emphasized the impact of both prenatal and postnatal health issues, finding that maternal infections and hypertension during pregnancy, as well as postnatal illnesses like asthma, chickenpox, and renal failure, were significantly associated with MIH [60]. Similarly, Ghanim et al. found that maternal psychological stress, frequent ultrasound exposure during late pregnancy, and high birth order increased the likelihood of MIH, while postnatal respiratory infections, unexplained high fevers, and pneumonia showed strong associations [61]. Koruyucu et al. expanded on these findings by identifying a range of prenatal, perinatal, and postnatal risk factors, including pregnancy complications, prematurity, frequent diarrhea, gastrointestinal diseases, renal failure, and childhood illnesses such as chickenpox and rubeola, all of which contributed to the development of MIH [62]. Wogelius et al. added nuance to this by showing that asthma drug use in children did not increase the overall prevalence of MIH, suggesting that certain medications may not always consistently impact enamel development [63]. Sonmez et al. also reported significant associations between MIH and prematurity, gastrointestinal problems, pneumonia, frequent high fevers, and childhood illnesses like measles and chickenpox [64]. Yet, factors such as low birth weight, maternal health, and vitamin or fluoride intake did not significantly correlate with MIH.

Elzein et al. focused on maternal and early childhood factors, finding that maternal consumption of canned food during pregnancy, early childhood fevers, and antibiotic use significantly increased MIH risk [65]. They reported that children whose mothers consumed canned foods during pregnancy were nearly three times more likely to develop MIH, and those who experienced high fevers or antibiotic use in early childhood had more than double the risk. Interestingly, Souza et al. concluded that no definitive etiological factor could be pinpointed for MIH, though they did observe that medical conditions like maternal anemia and early childhood respiratory issues such as rhinitis and bronchitis were more prevalent among children with MIH, suggesting a possible but inconclusive link [66].

The etiology of MIH is complex and multifactorial, involving a range of systemic and environmental factors. Respiratory illnesses, early childhood medical treatments such as antibiotics and aerosol therapy, and systemic conditions like celiac disease or HIV infection appear to play significant roles in MIH development. Prenatal factors, while not universally impactful, may interact with postnatal conditions to influence enamel hypomineralization. Ultimately, MIH appears to result from the interplay of genetic predispositions and early-life environmental and medical exposures.

A summary of the studies addressing the etiology of MIH is presented in Table 1.

**Table 1:** An overview of the studies addressing the etiology of MIH MIH: molar incisor hypomineralization; SNP: single nucleotide polymorphism

Author and year	Study design	Main findings
Hocevar et al. [45] 2020	Retrosoective study	Identified a significant association between the rs2245803 SNP in the *MMP20* gene and MIH susceptibility, particularly in homozygous individuals.
Bussaneli et al. [46] 2019	Retrospective study	Found a significant association between the rs10733708 polymorphism in the *TGFBR1* gene and severe MIH cases, highlighting gene-gene interactions between *AMELX* and immune response genes such as* IL4* and *IL17A*.
Teixeira et al. [47] 2017	Cross-sectional study	Higher concordance of MIH in monozygotic twins compared to dizygotic twins, suggesting a genetic influence on MIH.
Jeremias et al. [48] 2013	Retrospective study	Identified a significant link between the ENAM rs3796704 marker and MIH in Brazilian and Turkish populations, with additional associations with *AMELX* and *AMBN* genes affecting MIH severity.
Pang et al. [49] 2020	Case-control study	Found significant associations between polymorphisms in the *AMBN* and *MMP20 *genes and MIH susceptibility. Also identified gene-gene interaction involving *AQP5* gene.
Kühnisch et al. [50] 2013	Retrospective study	Identified a potential link between the *SCUBE1* gene on chromosome 22 and MIH, with specific SNPs showing significant association.
Durmus et al. [51] 2013	Case-control study	No significant correlation between prenatal conditions like bronchitis and MIH, but postnatal factors such as prolonged medication use and asthma were associated with MIH.
Wogelius et al. [52] 2010	Cross-sectional study	Children using Œ≤2-agonists and corticosteroids before age three had a higher prevalence of severe demarcated opacities, but no overall increased MIH risk from asthma drugs.
Shinde and Winnier [53] 2022	Case-control study	Aerosol therapy in early childhood increased MIH risk by 2.01 times, with early antibiotic use also contributing to higher risk.
Van der Tas et al. [54] 2018	Prospective cohort study	No significant association between vitamin D levels during fetal, neonatal, and childhood stages and MIH development by age six.
Costa-Silva et al. [55] 2013	Prospective cohort study	No significant correlations between deciduous molar hypominerlaization and MIH or between prenatal/perinatal conditions and MIH.
Kuklik et al. [56] 2020	Case-control study	Celiac disease increased MIH risk by 4.75-fold due to nutritional deficiencies from malabsorption.
Andrade et al. [57] 2016	Case-control study	A significantly higher prevalence of MIH in the HIV-infected group, with MIH being associated with the use of protease inhibitors in HIV-positive individuals.
Hysi et al. [58] 2016	Cross-sectional study	Children with a history of respiratory illnesses and antibiotic use during the first three years were more likely to develop MIH.
Tourino et al. [59] 2016	Cross-sectional study	Their findings indicated that MIH was significantly associated with early childhood illnesses, particularly asthma and bronchitis, as well as the use of antibiotics during the first four years of life.
Ahmadi et al. [60] 2012	Cross-sectional study	Significant association between prenatal and postnatal conditions, including maternal hypertension and infections, and postnatal illnesses such as asthma and MIH.
Ghanim et al. [61] 2012	Cross-sectional study	Maternal stress, frequent ultrasound exposure, and higher birth order increased MIH risk, along with postnatal conditions like respiratory infections.
Koruyucu et al. [62] 2018	Cross-sectional study	MIH was associated with pregnancy complications, prematurity, frequent diarrhea, and childhood illnesses such as chickenpox and rubeola.
Wogelius et al. [63] 2020	Cross-sectional study	No significant association between asthma drug use and MIH prevalence.
Sonmez et al. [64] 2013	Cross-sectional study	Prematurity, gastrointestinal issues, pneumonia, high fevers, and childhood illnesses like measles were significantly associated with MIH.
Elzein et al. [65] 2020	Case-control study	Maternal consumption of canned food during pregnancy, early childhood fevers, and antibiotic use increased MIH risk.
Souza et al. [66] 2013	Cross-sectional study	No specific etiological factor was definitively associated with MIH, although maternal anemia and respiratory issues were more prevalent in affected children.

**Figure 2:**
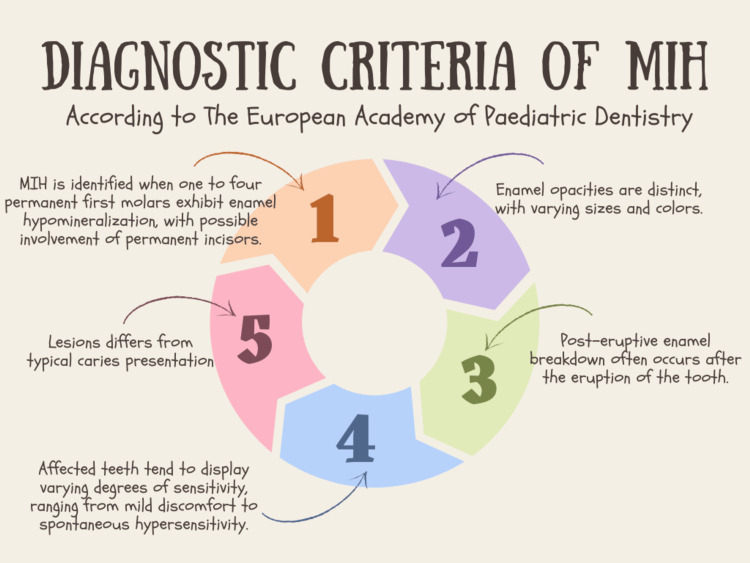
The diagnostic criteria of MIH according to The European Academy of Peadiatric Dentistry Image Credit: Ahmed Khalil (author); created with the help of www.canva.com Reference: [67]

The diagnostic criteria of MIH is depicted in Figure 2 [67].

Management

Flouride Varnish and Casein Phosphopeptide-Amorphous Calcium Phosphate (CPP-ACP)

In managing MIH, fluoride varnishes and casein-based products have become crucial preventive and therapeutic tools. Fluoride varnishes aid in remineralizing affected enamel, improving resistance to dental caries, and minimizing dentinal hypersensitivity by forming a temporary protective coating over hypomineralized regions. CPP-ACP products have shown potential in MIH treatment. CPP-ACP works by maintaining a supersaturated environment of calcium and phosphate ions on the tooth surface, promoting remineralization and preventing further enamel demineralization [68]. The combined use of fluoride varnishes and casein-based products offered a comprehensive approach to controlling MIH progression and reinforcing the structural integrity of affected teeth. Bakkal et al. demonstrated that both CPP-ACP and its fluoride-containing counterpart (CPP-amorphous calcium fluoride phosphate (ACFP)) significantly reduced enamel hypomineralization in children with MIH, suggesting that both treatments are effective in improving the mineral content of MIH-affected teeth over a short-term period [69]. Similarly, an in vitro study by Cardoso-Martins et al. confirmed the efficacy of CPP-ACP in enhancing enamel mineralization and structural organization in MIH lesions, reinforcing the potential of CPP-ACP as a useful tool in managing MIH [70]. Restrepo et al. examined the use of fluoride varnish in MIH-affected teeth but found no statistically significant improvement in enamel remineralization, casting doubt on the effectiveness of fluoride varnish alone for MIH lesions [71]. However, Sezer and Kargul demonstrated that combining calcium glycerophosphate with CPP-ACFP led to significant improvements in enamel mineralization in MIH-affected incisors, particularly in teeth with lower laser fluorescence scores, suggesting that a combined approach may yield better outcomes [72].

Nogueira et al. further explored the benefits of resin infiltration in preserving the structural integrity of MIH-affected teeth [73]. Their study showed that resin infiltration was more effective than fluoride varnish in reducing post-eruptive enamel, emphasizing the need for advanced techniques like resin infiltration in managing more severe cases of MIH. Similarly, Olgen et al. found that all tested remineralization agents improved the condition of MIH-affected teeth [74]. Yet, CPP-ACP and CPP-ACFP pastes offered faster and more pronounced results, particularly in yellow-brown defects. Biondi et al. provided a comparative analysis of various remineralizing agents, including 5% sodium fluoride varnish, sodium fluoride varnish with tricalcium phosphate, and CPP-ACP [75]. Results revealed that 5% sodium fluoride varnish with tricalcium phosphate was most effective for mild lesions, while 5% sodium fluoride varnish worked better for moderate lesions. Their findings suggested that the treatment choice should be tailored to the severity of MIH.

Research highlights the effectiveness of several remineralizing agents in managing MIH. While fluoride varnish alone may not be sufficient, combined treatments using agents like CPP-ACP, CPP-ACFP, and resin infiltration show significant potential in improving mineral content and protecting MIH-affected teeth from further damage.

Silver Diamine Fluoride (SDF)

Ballikaya et al. evaluated the impact of SDF in treating hypomineralized molars with early carious lesions in a 12-month clinical trial [76]. Both treatments demonstrated significant efficacy in reducing hypersensitivity and preventing enamel breakdown, with the silver-modified atraumatic restorative technique (SMART) showing higher survival rates on occlusal surfaces compared to palatal surfaces. However, their study noted a common side effect of SDF in the form of marginal discoloration, though this did not diminish its effectiveness for desensitization and caries prevention. Similarly, Al-Nerabieah et al. compared SDF and CPP-amorphous calcium phosphate fluoride varnish (ACPFV) [77]. The study found that SDF-treated molars experienced fewer new caries and higher rates of caries arrest compared to those treated with CPP-ACPFV. Both treatments were successful in preventing enamel breakdown and improving tooth sensitivity, indicating that SDF and CPP-ACPFV offer comparable efficacy in managing MIH-related issues. Building on this, Unverdi et al. conducted a randomized, split-mouth clinical trial comparing SDF and SMART [78]. While both treatments effectively reduced hypersensitivity and prevented further caries development, the SMART technique, which combines SDF with glass ionomer cement (GIC), demonstrated superior long-term caries prevention, with higher retention rates of the sealant. Despite differences in caries prevention outcomes, both approaches provided similar levels of desensitization.

Evidence highlights the efficacy of SDF and SMART in managing MIH. SMART shows some advantages in caries prevention due to its use of GIC. SDF, although effective, is associated with marginal discoloration, but this does not detract from its potential as a valuable tool for desensitization and caries management in MIH-affected teeth.

Sealant

The effectiveness of different types of sealants in MIH and maintaining the structural integrity of first permanent molars. Fragelli et al. evaluated the survival rates of sealants applied to the first permanent molars affected by MIH [79]. Their study demonstrated that MIH-affected molars had a comparable sealant survival rate to non-affected molars, with 72% of MIH-affected teeth maintaining their sealants, compared to 62% of the control group at the end of 18 months. This finding suggested that MIH-affected molars, despite their compromised enamel, can retain sealants effectively, highlighting the importance of sealant application for long-term protection in these cases. Building on this, Ozgur et al. compared the performance of resin-based and giomer-based fissure sealants on MIH-affected molars [80]. Their study revealed that resin-based sealants had significantly higher retention rates (68%) compared to giomer-based sealants (8%) after 12 months. This difference in performance was attributed to the superior bonding and retention properties of resin-based sealants, while the giomer sealants’ failure was likely due to the insufficient etching capacity of the self-etch primer used in the giomer group.

Together, these studies highlight that sealants are an effective treatment for MIH-affected molars. Yet, the type of sealant used plays a crucial role in treatment success. Resin-based sealants, in particular, offer better long-term retention and durability compared to giomer-based sealants, making them a more suitable option for managing MIH in clinical practice.

Restorative Treatment: Composite and Glass Ionomer Restorations

The management of MIH requires tailored restorative approaches, with various studies exploring the effectiveness of different materials and techniques to improve the long-term outcomes for affected teeth. Sonmez and Saat explored the effectiveness of composite resin restorations in MIH-affected molars, focusing on cavity preparation techniques and the use of sodium hypochlorite for deproteinization [81]. Their study found that removing hypomineralized tissue and using sodium hypochlorite to deproteinize the enamel significantly improved the retention and longevity of composite resin restorations, demonstrating the importance of thorough preparation for successful restorations in MIH cases. Building on this, Hakmi and Dashash compared direct and indirect composite restorations for treating MIH-affected molars [82]. Both techniques showed high clinical success rates over a 12-month period, with indirect restorations showing slightly better results and higher patient satisfaction due to shorter treatment times. This suggested that both techniques are effective. Yet, indirect restorations may offer more comfort and efficiency, particularly for pediatric patients. Yavuz et al. extended this research by assessing the performance of short fiber reinforced composite and glass hybrid restorations over a 36-month period [83]. Both materials showed comparable success rates in MIH-affected molars, although the overall success rate decreased over time. The study highlighted that both materials are effective for managing MIH when using selective caries removal techniques, though the left lower first molar was noted for higher failure rates, suggesting molar-specific considerations in long-term treatment planning. De Souza et al. [84] and Zahn et al. [85] further contributed to the understanding of adhesive restorations in MIH treatment. In the research by de Souza et al. [84], no significant differences were found between the self-etch and total-etch adhesive systems. Both systems demonstrated strong performance, with survival rates gradually decreasing over time, yet remaining satisfactory (68% for self-etching adhesive and 54% for total-etch adhesive at the end of 18 months). Similarly, the study by Zahn et al. examined the long-term effectiveness of direct resin restorations in MIH-affected teeth [85]. As in the previous study, no significant difference was observed between the survival rates of self-etch and total-etch systems. Additionally, both adhesive systems were successful in reducing sensitivity. Fragelli et al. focused on the use of GIC in MIH management, showing a high success rate of 78% after 12 months [86]. GIC was highlighted as a minimally invasive and effective treatment, particularly for younger patients, and the authors recommended using GIC to delay more invasive procedures until the child is mature enough for complex treatment.

Collectively, these studies emphasize the importance of selecting restorative materials and techniques that are carefully tailored to the severity of MIH and individual patient needs. While composite restorations and adhesive techniques offer promising solutions for managing MIH, GIC emerges as a particularly effective and minimally invasive option, especially for younger patients.

Crown Restorations

Oh et al. conducted a retrospective study that demonstrated the durability of preformed metal crowns, reporting a five-year survival rate of 82.8% [87]. However, the authors identified distal cavities and mandibular placement as significant risk factors for restoration failure, highlighting the importance of precise placement during the try-in stage to enhance longevity. Similarly, Geduk et al. conducted a prospective randomized trial comparing stainless steel crowns and zirconia crowns, with both showing high retention rates of 100% and 95.2%, respectively, over 18 months [88]. Stainless steel crowns proved highly durable and cost-effective. Yet, zirconia crowns offered better aesthetics and periodontal health, indicating the need to consider patient preferences and clinical outcomes when selecting crown materials.

Eldehna et al. compared zirconia and lithium disilicate overlays fabricated using computer-aided design and manufacturing technology in a randomized controlled trial [89]. Both materials demonstrated high success rates over 12 months, offering minimally invasive and effective solutions for restoring affected teeth. Dhareula et al. evaluated the performance of cast metal and resin onlays in children with severe cases of MIH [90]. Both materials showed retention rates of 95%, with metal onlays slightly outperforming resin in marginal adaptation and durability, suggesting their suitability for conservative yet reliable restorations.

Singh et al. conducted a 24-month trial comparing zirconia, lithium disilicate, and cast metal crowns [91]. All crown types showed excellent retention, complete resolution of hypersensitivity, and improved oral hygiene. However, zirconia and lithium disilicate crowns were preferred by parents and children for their aesthetic appeal. The findings reinforced the efficacy of full-coverage crowns, regardless of material type, in restoring severely hypomineralized molars.

These studies highlight that crown restorations, whether metal, zirconia, or resin-based, offer durable and effective solutions for managing MIH. Treatment should consider factors such as aesthetics, durability, periodontal health, and patient comfort, with future research focusing on long-term outcomes and advancements in restorative materials.

Laser Therapy

Effective management of sensitivity in MIH-affected teeth has become a clinical priority, with recent studies exploring innovative combinations of laser therapies and desensitizing agents to provide more sustained relief. Muniz et al. demonstrated that the combination of diode low-level laser therapy and fluoride varnish significantly reduced tooth sensitivity in children with MIH, with laser therapy providing immediate relief and fluoride varnish offering a more sustained reduction in sensitivity over time [92]. This combined approach outperformed either treatment used alone, showcasing its effectiveness in addressing sensitivity issues in MIH-affected teeth. Similarly, Machado et al. explored the use of neodymium-doped yttrium aluminum garnet (Nd:YAG) laser therapy alongside a desensitizing agent in a clinical case [93]. Their patient experienced immediate and long-lasting relief from hypersensitivity following this treatment, with the effects maintained over a one-month period. This case further supported the benefits of combining laser therapy with desensitizing agents for managing dentin hypersensitivity in MIH-affected teeth. Building on these findings, Zhao et al. conducted a randomized clinical trial to evaluate the combined use of erbium-doped yttrium-aluminum-garnet (Er:YAG) laser therapy and GLUMA desensitizer (Kulzer GmbH, Hanau, Germany) in reducing dentin hypersensitivity in children with MIH [94]. The study revealed that the combination of laser and desensitizer significantly outperformed either treatment alone in reducing hypersensitivity and improving the overall quality of life of affected children. The combination therapy not only resulted in greater pain relief but also improved oral health-related outcomes over a six-month period.

These studies suggest that combining laser therapies with desensitizing agents offers an effective and sustainable solution for managing dentin hypersensitivity in children with MIH. This approach provided immediate relief, making it a valuable strategy in clinical practice.

Extraction

When restoring the tooth is not feasible, and extraction becomes the only viable option, it is advisable to conduct an early orthodontic evaluation before extraction and closely monitor the development of the occlusion. Occlusal guidance is essential to ensure that the second permanent molars properly shift into the position of the extracted first molar. Brusevold et al. [95] demonstrated that maxillary permanent first molar extractions resulted in favorable spontaneous space closure with minimal need for orthodontic intervention, while mandibular first permanent molar often required additional orthodontic management. This distinction was echoed by Nordeen et al. [96], who found higher success rates of spontaneous space closure in the maxilla (82%) compared to the mandible (51%), particularly when extractions were performed between the ages of 8 and 10. The developmental stage of the second permanent molar also played a significant role, with extractions during developmental second molar stages D or E yielding higher success rates in both the maxilla and mandible. Ashley and Noar [97] further emphasized the importance of early extraction of compromised first permanent molar, recommending extraction between ages 8 and 10 to promote spontaneous space closure by second permanent molars and reduce the need for future orthodontic interventions. Their study also highlighted the presence and development of third molars as critical factors in achieving proper occlusion after mandibular first permanent molar extraction. Aldahool et al. [98] supported these findings, showing that timely extraction of first permanent molars before age 12, particularly when the first permanent molars were at developmental stages E or F, significantly increased the likelihood of successful space closure. Maxillary extractions had a higher success rate (94.1%) than mandibular extractions (74.1%), further emphasizing the favorable outcomes in the maxilla. Murphy et al. [99] expanded on the impact of mandibular first permanent molar extractions, revealing that such extractions encouraged mesial movement and uprighting of developing third molars, potentially improving their chances of a future eruption. This effect was more pronounced in children who underwent extraction compared to those who did not. Ciftci et al. [100] explored the role of prognostic factors influencing space closure following mandibular first permanent molar extraction. While developmental stage and age did not significantly correlate with successful space closure, the presence of third molars improved the likelihood of closure in the mandibular arch. Authors suggested that third molar development is a key factor in managing space following extractions.

Overall, these studies highlight the importance of timely intervention, particularly in the maxilla, and the developmental stages of second and third permanent molars in determining the success of spontaneous space closure after extraction of first permanent molars in MIH patients.

An overview of the studies addressing the management of MIH is depicted in Table 2.

**Table 2:** Summary of the studies addressing the management of MIH CPP: casein phosphopeptide; ACP: amorphous calcium phosphate; ACFP: amorphous calcium fluoride phosphate; MIH: molar incisor hypomineralization; SEM: scanning electron microscopy; SDF: silver diamine fluoride; SMART: silver-modified atraumatic restorative technique; ACPFV: amorphous calcium phosphate fluoride varnish; GIC: glass ionomer cement; Nd:YAG: neodymium-doped yttrium aluminum garnet

Author and year	Study design	Aim of the study	Main findings
Bakkal et al. [69] 2017	Pilot clinical study	Evaluate the impact of CPP-ACP and CPP-ACFP on noncarious MIH lesions.	Both treatments significantly reduced hypomineralization, no significant difference between the groups.
Cardoso-Martins et al. [70] 2022	In-vitro study	Assess the efficacy of CPP-ACP in the management of MIH lesions using Raman spectroscopy and SEM.	Significant improvement in enamel mineral density and structure for MIH lesions with CPP-ACP.
Restrepo et al. [71] 2017	Clinical trial	Investigate the effect of fluoride varnish on enamel remineralization in MIH-affected teeth.	No significant improvement in remineralization of MIH lesions after fluoride varnish application.
Sezer and Kargul [72] 2022	Randomized controlled clinical trial	Evaluate the efficacy of calcium glycerophosphate and CPP-ACFP in remineralizing MIH-affected incisors.	Both treatments improved mineralization, with CPP-ACFP showing the best results.
Nogueira et al. [73] 2021	Randomized controlled trial	Investigate the effectiveness of fluoride varnish and resin infiltration in preserving MIH-affected teeth over 18 months.	Resin infiltration was more effective than fluoride varnish in preventing post-eruptive enamel breakdown.
Olgen et al. [74] 2021	Randomized controlled trial	Compare the effectiveness of various remineralization agents in managing MIH defects.	CPP-ACP and CPP-ACFP show faster effects.
Biondi et al. [75] 2017	Clinical trial	Compare 5% sodium fluoride varnish (Duraphat), 5% sodium fluoride varnish with tricalcium phosphate (Clinpro), and casein phosphopeptide-amorphous calcium phosphate (Recaldent)	5% sodium fluoride varnish with tricalcium phosphate was most effective for mild lesions, while 5% sodium fluoride varnish worked better for moderate lesions.
Ballikaya et al. [76] 2021	Clinical trial	Evaluate the effectiveness of SDF and SMART in managing early carious lesions in hypomineralized molars.	Both treatments reduced hypersensitivity and prevented enamel breakdown.
Al-Nerabieah et al. [77] 2023	Randomized controlled trial	Compare SDF and casein phosphopeptide-amorphous calcium phosphate fluoride varnish CPP-ACPFV in preventing enamel breakdown and improving tooth sensitivity in MIH-affected molars.	SDF-treated molars had fewer new caries and higher caries arrest rates than CPP-ACPFV.
Unverdi et al. [78] 2024	Randomized split-mouth clinical trial	Compare SDF and SMART in reducing hypersensitivity and preventing caries in MIH-affected permanent molars.	Both treatments reduced hypersensitivity, with SMART showing better caries prevention and retention rates.
Fragelli et al. [79] 2017	Clinical trial	Evaluate the survival rate of sealants applied to first permanent molars (FPMs) affected by MIH.	Sealants on MIH-affected molars had a similar survival rate to non-affected molars, with 72% survival at 18 months.
Ozgur et al. [80] 2022	Randomized clinical trial	Compare the clinical performance of resin-based and giomer-based sealants on molars affected by MIH.	Resin-based sealants showed significantly higher retention rates (68%) than giomer-based sealants (8%).
Sonmez and Saat [81] 2017	Clinical trial	Assess the performance of composite resin restorations in MIH-affected molars using different cavity designs and deproteinization techniques.	Removing hypomineralized tissue and using sodium hypochlorite improved the success and longevity of restorations.
Hakmi and Dashash [82] 2023	Randomized controlled trial	Compare the clinical performance of direct and indirect composite restorations for treating molars affected by MIH.	Both direct and indirect composite restorations showed high clinical success rates, with indirect restorations offering higher patient satisfaction.
Yavuz et al. [83] 2024	Randomized split-mouth study	Assess the clinical performance of short fiber reinforced composite and glass hybrid restorations in MIH-affected molars and carious lesions.	Short fiber reinforced composite and glass hybrid restorations had similar success rates, though failure rates were higher for the left lower first molar.
De Souza et al. [84] 2017	Randomized controlled trial	Evaluate the 18-month clinical performance of composite resin restorations in molars affected by MIH, comparing self-etch and total-etch adhesive systems.	Both adhesive systems were effective, with no significant differences in survival rates at 18 months. Conservative cavity preparation improved outcomes.
Zahn et al. [85] 2021	Randomized controlled trial	To assess the survival of adhesive restorations in MIH-affected first permanent molars, evaluating self-reported dental pain and anxiety.	Both adhesive systems were effective, with no significant differences in survival rates at 12 months. Both protocols reduced sensitivity.
Fragelli et al. [86] 2021	Clinical trial	Assess the clinical performance of GIC restorations in managing MIH over a 12-month period.	GIC showed a 78% success rate at 12 months, maintaining tooth structure, and was recommended as a minimally invasive treatment.
Muniz et al. [92] 2019	Randomized Clinical Trial	Assess the efficacy of diode low-level laser therapy combined with fluoride varnish in managing tooth sensitivity in MIH.	Combined laser therapy and fluoride varnish provided better results in reducing sensitivity than either treatment alone.
Machado et al. [93] 2019	Clinical case	Explore the combined use of Nd:YAG laser and a desensitizing agent for managing dentin hypersensitivity in MIH.	Immediate and sustained relief from sensitivity noted, with improvements after one week and maintained at one month.
Zhao et al. [94] 2023	Randomized clinical trial	Evaluate the effectiveness of laser therapy combined with GLUMA desensitizer in reducing dentin hypersensitivity in MIH-affected children.	Laser and GLUMA desensitizer significantly reduced hypersensitivity and improved oral health-related quality of life.
Brusevold et al. [95] 2022	Retrospective study	Assess spontaneous space closure following the extraction of first permanent molars severely affected by MIH.	Favorable spontaneous space closure in maxilla, mandibular extractions showed less favorable outcomes requiring orthodontic intervention.
Nordeen et al. [96] 2022	Retrospective study	Evaluate factors associated with spontaneous space closure of second permanent molars following early extraction of first permanent molars.	Higher success rates for space closure in maxilla; extraction age and developmental stage were critical predictors of outcomes.
Ashley and Noar [97] 2019	Review	Evaluate the management of compromised first permanent molars and benefits of early extraction to promote spontaneous space closure.	Early extraction between ages 8-10 recommended to minimize orthodontic intervention.
Aldahool et al. [98] 2024	Retrospective study	Assess the success rate of spontaneous space closure after early extraction of first permanent molars in children.	Most successful in the maxilla; extractions at specific developmental stages E/F yielded the best results.
Murphy et al. [99] 2022	Retrospective study	Evaluate the impact of extracting lower first permanent molars on development and positioning of mandibular third molars in children.	Extraction encouraged mesial movement and uprighting of third molars, improving chances of eruption.
Ciftci et al. [100] 2021	Cross-sectional study	Evaluate prognostic factors influencing spontaneous space closure after extraction of the first permanent mandibular molar in children.	The presence of third molars improved the likelihood of successful space closure in the mandibular arch.

Future directions

The precise cause of MIH remains uncertain, largely due to the numerous potential factors involved and the reliance on retrospective study designs in most of the existing research. Future directions in the management and understanding of MIH should focus on advancing both research and clinical practices. A deeper exploration of genetic and epigenetic factors could help understand the complex etiology of MIH, potentially leading to more targeted preventive strategies. Additionally, long-term clinical studies are needed to evaluate the effectiveness and durability of current treatment approaches in the long term. There is also a growing need for patient-centered research, including studies on the psychosocial impact of MIH and its influence on oral health-related quality of life. Moreover, early diagnostic tools and more individualized treatment plans based on severity and patient-specific factors could improve outcomes. As technology and materials evolve, innovative solutions such as bioactive materials and minimally invasive techniques may play an increasingly important role in the future management of MIH.

Limitations

Several limitations of this review are recognized. A notable limitation is that the review was predominantly limited to English publications, potentially overlooking relevant studies available in other languages. The information used in these studies was often collected through questionnaires or interviews, which depend on individual memory and can result in inaccuracies. Such methods introduce bias and the results are generally affected. Despite these limitations, this review provides valuable insights for a better understanding of the condition.

## Conclusions

The etiology of molar incisor hypomineralization is multifactorial, involving a complex interplay of prenatal, perinatal, postnatal, environmental, and potentially genetic factors. The origins of the condition are not yet fully understood, and the retrospective nature of most studies makes it difficult to establish clear causal relationships. The main goals of the treatment of MIH are to alleviate dental sensitivity, preserve remaining enamel, and improve aesthetics. Management strategies must be tailored to each patient, taking into account the severity of the condition and the individual needs.
